# Effects of season, depth and pre-cultivation fertilizing on *Ulva* growth dynamics offshore the Eastern Mediterranean Sea

**DOI:** 10.1038/s41598-023-41605-4

**Published:** 2023-09-07

**Authors:** Meiron Zollmann, Alex Liberzon, Ruslana R. Palatnik, David Zilberman, Alexander Golberg

**Affiliations:** 1 Porter School of Environmental and Earth Sciences, Tel Aviv University, Tel Aviv, Israel; 2 School of Mechanical Engineering, Tel Aviv University, Tel Aviv, Israel; 3 Department of Economics and Management, The Max Stern Yezreel Valley College, Afula, Israel; 4 NRERC-Natural Resource and Environmental Research Center, University of Haifa, Haifa, Israel; 5 Department of Agricultural and Resource Economics, The University of California at Berkley, Berkeley, CA USA

**Keywords:** Environmental biotechnology, Marine biology

## Abstract

Offshore macroalgae production could provide an alternative source of biomass for food, materials and energy. However, the offshore environment in general, specifically the Eastern Mediterranean Sea (EMS) offshore, is a high energy and low nutrients environment, thus challenging for macroalgae farming. In this study, we experimentally investigated the impact of season, depth, and pre-cultivation fertilization duration on the growth rates and chemical composition of offshore *Ulva* biomass, and developed a predictive model tailored to offshore conditions, capable of estimating both biomass growth rate and nitrogen content. Specifically, we measured *Ulva* biomass growth rate and internal nitrogen in the nitrogen-poor EMS a few kilometers offshore the Israeli coast at various depths and on-shore pre-cultivation fertilization schedules. Based on these data, we constructed a predictive cultivation model of *Ulva* offshore growth, which allows for the optimization of fertilization requirements for offshore cultivation. This study provides new insights on the effects of seasonality, depth, and pre-cultivation fertilization duration on growth rates and chemical composition of offshore *Ulva* sp. biomass production.

## Introduction

Offshore-grown macroalgae provide a sustainable biomass source for biorefineries to produce proteins, platform chemicals, and energy without any need for arable land or fresh water^1^. However, macroalgae still present only a tiny percent of the global biomass supply of ~ 30 × 10^6^ ton Fresh Weight (F.W.) of macroalgae compared to 16 × 10^11^ tons of terrestrial crops, grasses, and forests^2–5^. Green macroalgae from the *Ulva* species is interesting biomass for biorefinery as it grows globally, and it was already converted to protein, cellulose, starch, ulvan (bioactive polysaccharide), fatty acids, minerals, biocrude, biochar, ethanol, biogas^6^, and polyhydroalkanolyes^7^, all of which could serve as building blocks for a sustainable bioeconomy^8^. Although traditionally grown onshore, a recent study demonstrated also the feasibility of *Ulva* cultivation offshore, in a sheltered environment^9^.

However, production of the *Ulva* biomass, in an open-water offshore environment that is characterized with high wave energy and low nutrients concentrations is challenging^10–15^. Artificial fertilizing is generally not recommended but can be performed via integration to fish farms in an Integrated Multitrophic Aquaculture (IMTA)^16^ or artificial upwelling of nutrient-rich deep water^13^. Another potential fertilizing solution, although logistically challenging, is to utilize *Ulva*’s high uptake rates (up to 470 μmol N g^−1^ D.W. h^−1^)^17–21^ and recharge critical nutrients by rapid fertilizing in a designated fertilizing tank, which can be performed on or offshore. Theoretically, onshore fertilizing can be added to other pre-cultivation activities that are performed traditionally in the onshore nursery, such as seedling propagation and rope seeding.

Given the intricate nature and advanced operational logistics inherent in offshore environments, establishing expansive offshore farms for biomass production necessitates meticulous planning and careful consideration of numerous design and operational parameters. Factors such as cultivation depth, the duration of the cultivation cycle, and pre-cultivation fertilization must be determined with a comprehensive understanding of seasonal growth dynamics and environmental impacts. These factors are pivotal in ensuring the economic viability of production.

Experience from terrestrial agriculture shows that dynamic models that aim to predict the field yield significantly improve food supply systems economics and long-term sustainability^22^. Such detailed models, which combine biomass productivity, crop yields, sustainability, and economics, still need to be made available for *Ulva* macroalgae offshore farms. Yet, initial steps in this direction have been made by developing growth function models to predict seasonal blooms^23–34^ and biomass productivity in the controlled photobioreactors^35–39^. The existing models for offshore biomass productivity of the *Ulva* species do not provide information on the effects of dynamic environmental factors such as light, temperature, and nutrients on biomass productivity and chemical composition. Thus, their applicability for the design of real-time seaweed farms is low^40, 41^. A more advanced dynamic macroalgae productivity model was published only recently^42^, but it focused on red and brown seaweed and did not relate to the seasonal effects and to the varying nitrogen content in the biomass.

This study aims to comprehend the influence of seasonality, depth, and pre-cultivation fertilization duration on the growth rates and chemical composition of open-water offshore *Ulva* biomass production. Additionally, we sought to develop a predictive model for estimating biomass offshore productivity and internal nitrogen concentration. To achieve these objectives, we conducted experiments in the nitrogen-poor (oligotrophic) Eastern Mediterranean Sea (EMS), a few kilometers offshore the Israeli coast, considering various depths and pre-fertilization regimes. Drawing upon the data obtained from these experiments, we constructed an accurate predictive model for biomass productivity, specifically adapted to offshore conditions.

## Results

**Figure 1:**
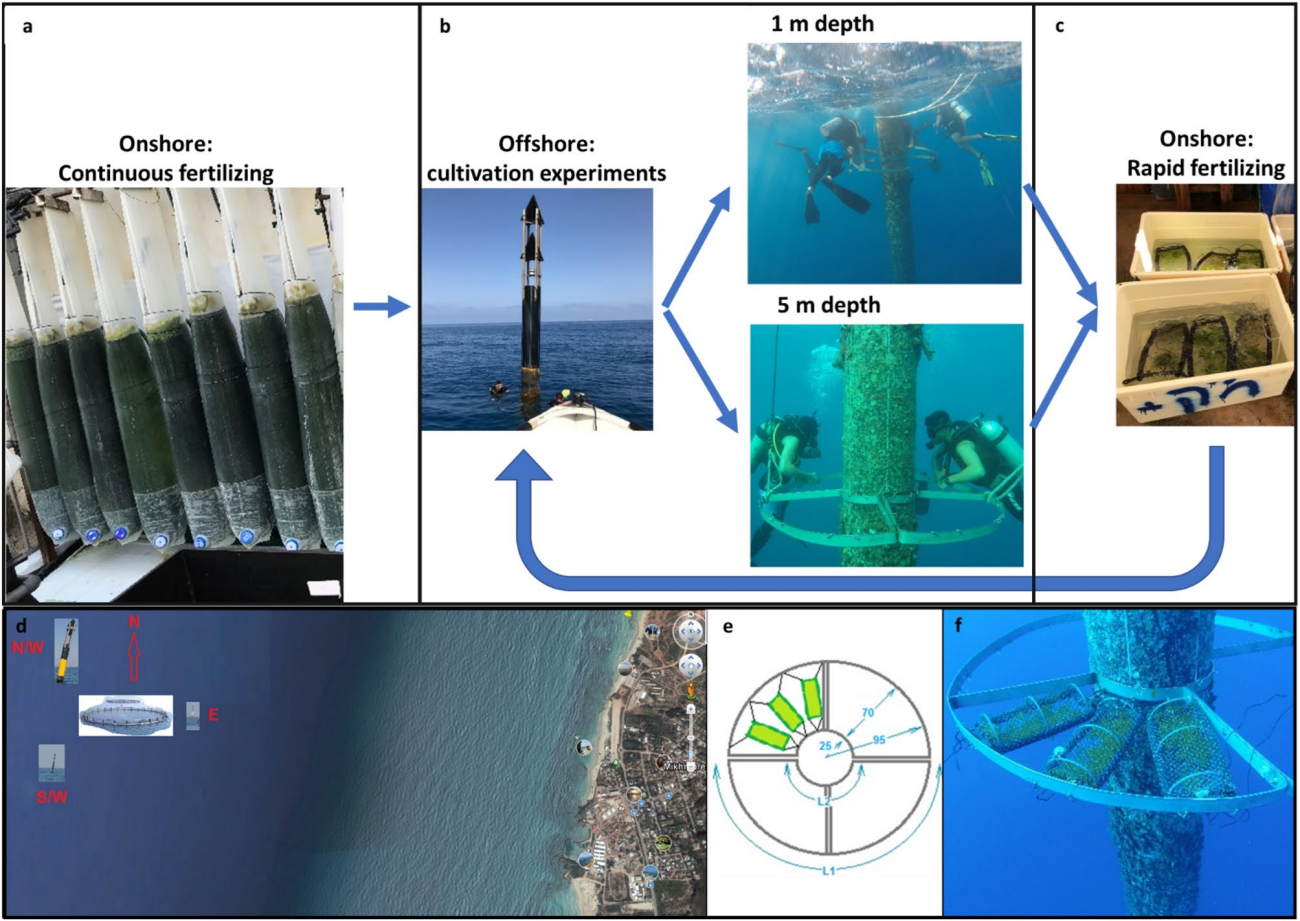
Top row: experimental flow diagram. **(a)** Image of the onshore continuous fertilizing of the *Ulva* sp. stock in an MPBR system in the aquaculture center in Michmoret, **(b)** images of: the offshore experiments site on the North-Western marking buoy of the Lev-Yam fish cages and the installation of the cultivation rings in a depth of 1m and in a depth of 5m, **(c)** onshore rapid fertilizing in aerated tanks, used between offshore cultivation periods. Bottom row: illustration images: **(d)** map of cultivation site offshore Michmoret,** (e)** illustration of a single cultivation ring and attached cages, and **(f)** three cages stocked with *Ulva* biomass installed on the cultivation system.

The results comprise of (a) experimental results of growth rates and chemical compositions of *Ulva* sp. macroalgae cultivated in cages offshore the EMS under different conditions, and (b) model simulations for *Ulva* sp. growth rates and chemical compositions. A scheme of the cultivation experiments is presented in Fig. 1. *Ulva* sp. cultivation experiments in offshore cages provide year-round growth rates and chemical compositions in the Israeli EMS in two depths (1 and 5 m, Fig. 1b). In addition, it examines the effectiveness of short-term fertilizing treatments (Fig. 1c) between two offshore low-nutrient cultivation periods. All experiments followed a similar design, validating the cultivation model under naturally varying environmental conditions offshore. The full results of Daily Growth Rate (DGR) and internal N in the different experiments are summarized in Tables 1 and 2. Data analysis was performed step-by-step, starting with the effect of cultivation depth, continuing with the effect of pre-cultivation fertilization regime (continuous vs. rapid), and finishing with the effect of cultivation date.

**Table 1:** Daily growth rates measured for *Ulva* sp. in different offshore cultivation experiments.

	Dates	Fertilizing regime*	Depth	N (# samples)	Mean DGR (% day^−1^)	S.D. (% day^−1^)	S.E. (% day^−1^)	95% conf. interval (% day^−1^)	
Preliminary experiment	29.5.19–6.6.19	Rapid	1	4	−25.6	11.3	5.7	−43.6	−7.6
			5	6	−39.4	47.5	19.4	−89.2	10.4
Experiment 1	22–29.7.19 30.7.19–5.8.19	Continuous	1	6	1.2	3.2	1.3	−2.2	4.6
			5	6	9.8	2.4	1	7.2	12.4
		Rapid	1	6	−19.3	11.1	4.6	−31	−7.6
			5	6	−11.7	5.4	2.2	−17.4	−5.9
Experiment 2	30.10.19–6.11.19	Continuous	1	9	−4.3	4.5	1.5	−7.7	−0.8
			5	9	6.1	0.8	0.3	5.4	6.7
Experiment 3	11–18/23.12.19	Continuous	5	12	0.6	3.1	0.9	−1.3	2.6
Experiment 4	30.4.20–12.5.20	Continuous	5	12	7.3	2.4	0.7	5.8	8.8

*Fertilizing regime relates to nutrient enrichment prior to the cultivation experiment. Continuous fertilizing refers to prolong cultivation in the outdoor MPBR in Michmoret which receives nutrient rich near-shore water, whereas rapid fertilizing refers to a 18–24 h of onshore fertilizing between two cultivation periods in the nutrient poor offshore environment.

**Table 2:** Internal N measured for *Ulva* sp. in different experiments of the offshore cultivation experiment.

	Dates	Fertilizing regime*	Depth	N (# samples)	Mean internal N (% g N g^−1^ DW)	S.D. (% g N g^−1^ DW)	S.E. (% g N g^−1^ DW)	95% conf. interval (% g N g^−1^ DW)	
Preliminary experiment	20–28.5.19 29.5.19–6.6.19	Continuous	1	1	0.84	–	–	–	–
			5	2	0.63	0.16	0.11	−0.78	2.03
		Rapid	1	1	0.83	–	–	–	–
			5	2	0.50	0.05	0.04	0.01	0.98
Experiment # 1	22–29.7.19 30.7.19–5.8.19	Continuous	1	2	0.91	0.27	0.19	−1.52	3.34
			5	2	0.83	0.10	0.07	−0.06	1.72
		Rapid	1	2	0.72	0.08	0.06	−0.02	1.47
			5	2	0.68	0.11	0.08	−0.30	1.67
Experiment # 2	30.10.19–6.11.19	Continuous	1	2	0.98	0.01	0.01	0.89	1.07
			5	2	1.05	0.18	0.13	−0.57	2.66
Experiment # 3	11–18/23.12.19	Continuous	5	8	1.95	0.28	0.10	1.72	2.18
Experiment # 4	30.4.20–12.5.20	Continuous	5	5	1.64	0.13	0.06	1.48	1.81

*Fertilizing regime relates to nutrient enrichment prior to the cultivation experiment. Continuous fertilizing refers to prolong cultivation in the outdoor MPBR in Michmoret which receives nutrient rich near-shore water, whereas rapid fertilizing refers to a 18–24 h of onshore fertilizing between two cultivation periods in the nutrient poor offshore environment.

### Cultivation depth

**Figure 2:**
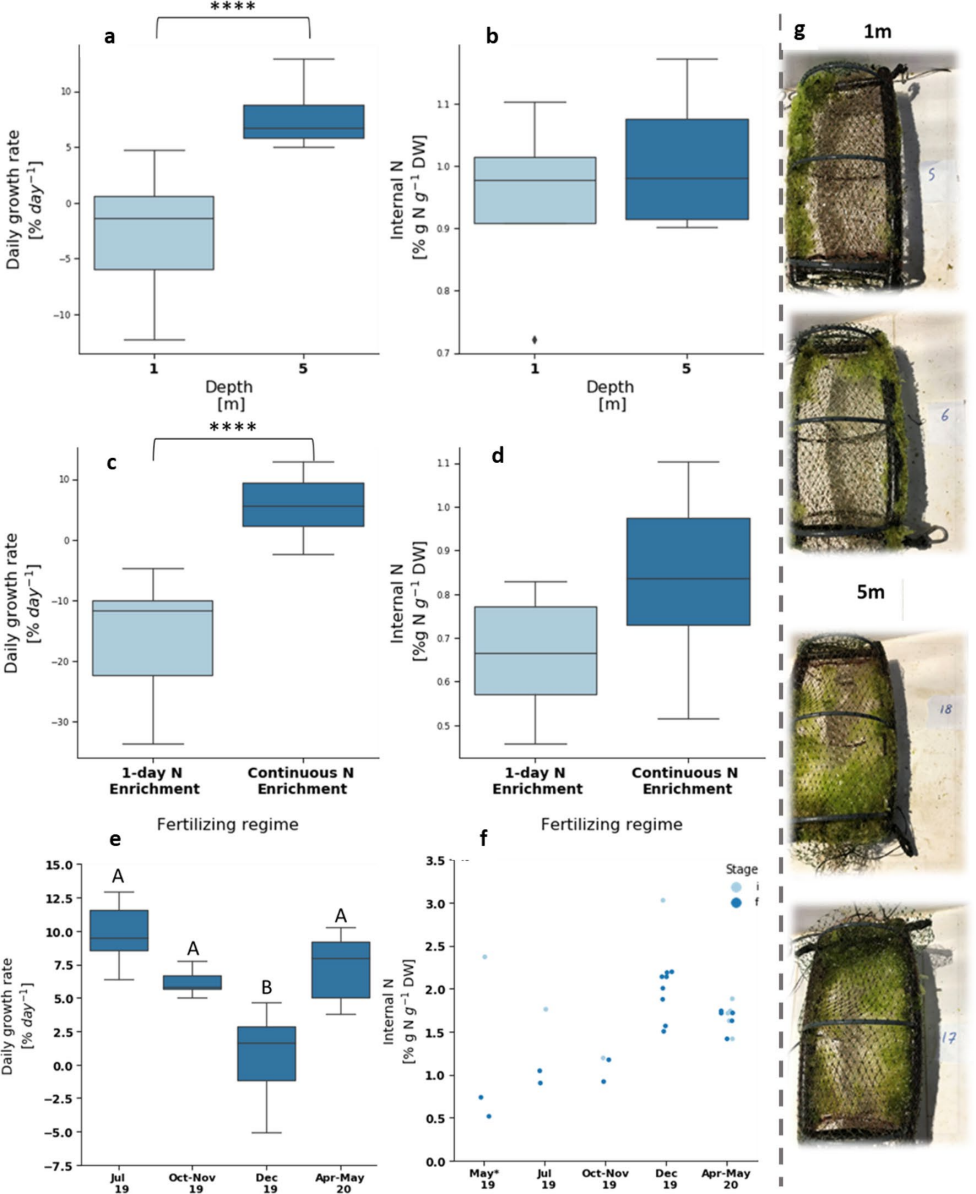
Experimental results of daily growth rate (**a**,**c,e**) and internal N (**b**,**d**,**f**) of *Ulva* sp. macroalgae cultivated in cages offshore the EMS under different conditions of depth (top row), pre-cultivation fertilizing (middle row) and experiment date (bottom row). Representative images of cages with *Ulva* sp. biomass after cultivation in depths of 1 m (top) and 5 m (bottom) are presented in the right column **(g)**. Top row: 1 m depth (light blue) vs 5 m depth (dark blue). Analysis included only *Ulva* sp. cultivated after continuous nutrient enrichment in experiments #1 and #2. Sample sizes: 15 for DGR and 4 for internal N for each depth. Asterisks indicate statistical significance of difference with ****p < 0.0001, calculated by the two-tailed Mann–Whitney U test. Middle row: rapid (1-day) pre-cultivation nutrient enrichment (light blue) vs continuous pre-cultivation nutrient enrichment (dark blue). Sample sizes: rapid nutrient enrichment: 22 for DGR and 7 for internal N. continuous nutrient enrichment: 54 for DGR and 21 for internal N. Asterisks indicate statistical significance of difference with ****p < 0.0001, calculated by the two-tailed Mann–Whitney U test. Bottom row: DGR **(e)** and internal N **(f)** at different experiment dates. On the left, Group A is statistically different from group B, in a significance of p < 0.05, calculated by the post-hoc Dunn’s test with the Bonferroni adjustment method for pairwise comparison. On the right, light blue dots present internal N at the beginning of each experiment and dark blue dots represent internal N at the end of each experiment. Analysis included only *Ulva* sp. cultivated after continuous nutrient enrichment in a depth of 5 m. From the preliminary run (May 19) we show only internal N results, as the DGR measurements are meaningless due to biomass losses. Sample sizes: 6–12 for DGR and 2–8 for internal N for the different experiments.

Cultivation of *Ulva* sp. offshore the EMS in a depth of 5 m has shown higher growth rates (p-value < 0.0001, N = 30) than cultivation in a depth of 1 m, but similar internal N values (N = 8). We compare (Fig. 2a, b) the results of experiment #1 and experiment #2, with cultivations at both depths. We attribute the results to different environmental conditions, specifically waves and currents. Visual observations (Fig. 2g) indicate an essential difference between depths. Whereas in the 5 m cages, the biomass is distributed throughout the whole surface of the cages, in the 1 m cages, the biomass is clumped on the cage frame. This distribution difference indicates that the water motion, produced by waves and currents, is more prominent at the 1 m depth. Based on this observation, we suggest two potential mechanisms causing lower growth rates in the 1m depth: (1) faster mechanical wearing and biomass losses in the shallower depth, and/or (2) biomass clumping inhibited growth due to a smaller surface area and a lower exposure to light.

### Fertilizing regime

One day (rapid) onshore fertilizing method effectivity is compared to that of continuous nutrient enrichment method using the DGR and internal N at the end of the cultivation period. Results from experiment #1 show higher DGR (p-value < 0.05, Fig. 2c) and slightly higher internal N for continuous fertilization (Fig. 2d). Figure S2 signifies the result comparing the results of each depth (1 and 5 m cages) separately (p-value < 0.001). In contrast, both depths have a consistent but relatively mild effect on internal N. In the rapid fertilizing experiments, no difference can be found between DGR of samples fertilized in regular seawater and samples fertilized in enriched seawater. Based on these results, we decided to focus on the experiments performed after continuous nutrient enrichment in the Macroalgae Photobioreactor (MPBR) instead of the rapid onshore fertilizing that was proven ineffective in these conditions.

### Seasonal effects

We compare here results from four experiments from only 5 m depth experiments with continuous fertilization method. Growth rates vary seasonally (Fig. 2e) (p-value < 0.0001). Multiple comparison tests found that growth rates in December 2019 were significantly lower (and in some cases negative) than all other experiments that are comparable in DGR (p-value < 0.05).

We study weather conditions that led the DGR and internal N at different experiments, specifically irradiance, rain, waves, and currents (Suppl. Appendix C). The low growth in the December experiments is explained by some combination of stormy weather: rough sea conditions, and light limitation. The first few days of the December cultivation period were stormy, with a significant wave height (highest third of the waves) higher than 2.5 m (measurements from the Hadera GLOSS #80 station). Four cages show biomass losses, probably a result of the waves. The biomass at 5 m depth is also clumped on the cage frame in these conditions, similar to the 1 m cages presented above (Fig. 2e). The stormy days also induce light limitation through two different mechanisms: clouds reduce global irradiance at the sea surface and significant rain events on December 12 (28 mm per day, based on IMS data). Such significant rain events typically cause flash floods in the Alexander estuary, resulting in coastal enrichment of particulate matter and increased turbidity. Notably, during the April–May cultivation period, the significant wave height was relatively high (around 2 m), and some rainfall was measured (5 mm during 4–5 May), but the growth rate was high. A prominent difference between various high-wave periods is the wind direction: western winds during December versus Eastern winds during May. Although it is impossible to pinpoint the dominant mechanism, we believe that the high turbidity and strong waves are equally probable. In the winter experiment (December 19), half of the cages were harvested right after the storm and half remained offshore for longer. The cages harvested right after the storm had a larger portion of biomass loss compared to the cages that stayed for another week (three cages versus one), pointing to the biomass recovery during the additional five days. Higher irradiance (i.e., fewer clouds and lower turbidity) and lower waves enabled the biomass to grow and accumulate despite initial losses. Internal N in the longer cultivation duration slightly decreased, consistent with the growth rates in a nutrient-poor environment. We conclude that winter growth is possible but may require longer cultivation periods, especially after storms and rainfall. Such more extended cultivation periods are possible if the nutrients are sufficient to keep the internal N around 1% g N g^−1^ D.W. (Fig. 2f).

Internal N decreases during the cultivation experiments with respect to the initial internal N (taking into account also the results of the preliminary experiment) (Fig. 2f, p-value < 0.01). Higher DGR leads to lower internal N in experiments #1–4 (Pearson’s r = −0.462), supporting the growth model hypothesis that in an oligotrophic environment growth is based mostly on initial internal N. However, there are unusually high internal N results in experiments #2 and #4 with respect to the high DGR, suggesting that there was an external additional nutrient supply to the cultivation site during these periods. This suggestion is also supported by the mass balance presented in Table 3, confirming that N uptake was substantial in those two periods, especially during April–May 2020. We attribute this supply to the sea currents from the rich nutrient site of Alexander Estuary (see “Discussion” for further discussion).

**Table 3:** Nitrogen mass balance in offshore experiments.

Run #	Dates	Total initial N per cage* (g N)	Total final N per cage* (g N)		Sample #	Trend
			Average	STD		
1	22–29.7.2019	0.05	0.05		2	–
2	30.10–6.11.2019	0.04	0.05		2	Minor increase
3	11–23.12.2019	0.09	0.06	0.03	8	Decrease
4	30.4–12.5.2020	0.06	0.12	0.03	5	Significant increase

*Calculated by multiplying dry weight by N content (% g N g^−1^ D.W.).

**Data taken only from experiments after continuous fertilizing from 5 m depth.

### Offshore cultivation simulations

The main purpose of the mathematical model and the associated software^54^ (is to estimate the external N supply to the offshore cultivation sites and predict growth rates and internal N conditions for optimal cultivation cycles. Model parameters, that were determined in the preliminary calibration process, are detailed in Table 4. The analysis is of the results of cultivation in a depth of 5 m after continuous fertilization in experiments #1, 2, and 4. Experiment #3 (December 2019) is excluded from the model due to a storm-related biomass loss.Additional factors that we considered are the current regime, potentially changing external N concentration (${N}_{ext}$), and the wave height, affecting biomass distribution inside the cages. To alleviate the effect of unequal biomass distribution after the high wave event experienced on May 6 (Table 5), we assumed that the surface area covered by *Ulva* decreased by a factor of 2 after the high waves. Considering both factors, we assumed that the initial N concentration in the sea was similar to the background concentration when the dominant current direction was North-Easterly (i.e. 0.75 µM N, as was estimated for experiment #1), and that it increased following the change of current direction from Northly to Westerly.

**Table 4:** Model parameters.

Parameter #	Parameter symbol	Definition	Calibration system	Value	Examined range	Unit
1	${\widehat{\upmu }}_{max}$	Maximum specific growth rate at S = 39PSU	Based on previous measurements^43^	0.03		Light h^−1^
2	${T}_{min}$	Minimal temperature		4		$^\circ{\rm C}$
3	${N}_{int min}$	Minimum internal N concentrations in *Ulva*		0.48		% g N g^−1^ DW
4	$\widehat{\lambda }$	Biomass specific losses rate as at S = 39PSU	Indoor controlled photobioreactor^43^	0.003	0.001–0.005	Light h^−1^
5	${N}_{int max}$	Maximum internal N concentrations in *Ulva*		4.5	4.5–5	% g N g^−1^ DW
6	${N}_{int crit}$	Critical internal N concentration		0.7	0.7–3.2	% g N g^-1^ DW
7	${K}_{s}$	N half-saturation uptake constant		10	10–30	μmol N L^−1^
8	${V}_{max}$	Maximum N uptake rate		50	50–250	μmol N g^−1^ DW h^−1^
9	${K}_{I}$	Light half-saturation constant		15	0.1–3	μmol photons m^−2^ s^−1^
10	${K}_{0}$	Light extinction coefficient in the water		0.2	0.01–0.2	m^−1^
11	${S}_{min}$	Minimal salinity	Outdoor semi-controlled bottles photobioreactor (Suppl Appendix A)	0	0–10	PSU
12	${S}_{opt}$	Optimal salinity		28	10–35	PSU
13	${S}_{max}$	Maximal salinity		50	40–50	PSU
14	${T}_{opt}$	Optimal temperature		18	15–25	$^\circ{\rm C}$
15	${T}_{max}$	Maximal temperature		36	31–37	$^\circ{\rm C}$
16	n	Temperature function exponent		5.1	1–6	$-$
17	${K}_{a}$	*Ulva* light extinction coefficient	Offshore system	0.2	0.01–0.4	m^2^ g^−1^ DW

**Table 5:** Sea conditions, model errors and estimated offshore N concentrations during the offshore experiments.

Experiment #	Dates	Dominant currents	Waves (m)	Estimated ${N}_{ext}$ (µM N)	Measured/modeled m (g FW L^−1^) (# samples)	Measured/modeled ${N}_{int}$ (% g N/g DW) (# samples)	Mean RMSRE (%)
1	22–29.7.2019	North-Easterly	< 1	0.75	4.05 ± 0.57/3.7 (5)	0.83/0.84 (2)	27.1
2	30.10–6.11.2019	Westerly	< 1	1.25	3.15 ± 0.17/3.25 (9)	1.05/1.05 (2)	18.1
4	30.4–12.5.2020	30.4–7.5: North-Easterly 8–12.5: Westerly	30.4–5.5: < 1 6.5: ~ 2.5 7–12.5: < 1.5	30.4–7.5: 0.75 8–12.5: 3.6	5.01 ± 1.21/7.3* (12)	1.64 ± 0.12/0.78* (5)	42.8*

*Model predictions with a constant ${N}_{ext}$ of 0.75 µM N are 7.6 g FW L^−1^, 0.78% g N/g DW and mean RMSRE of 45.1%.

**Figure 3:**
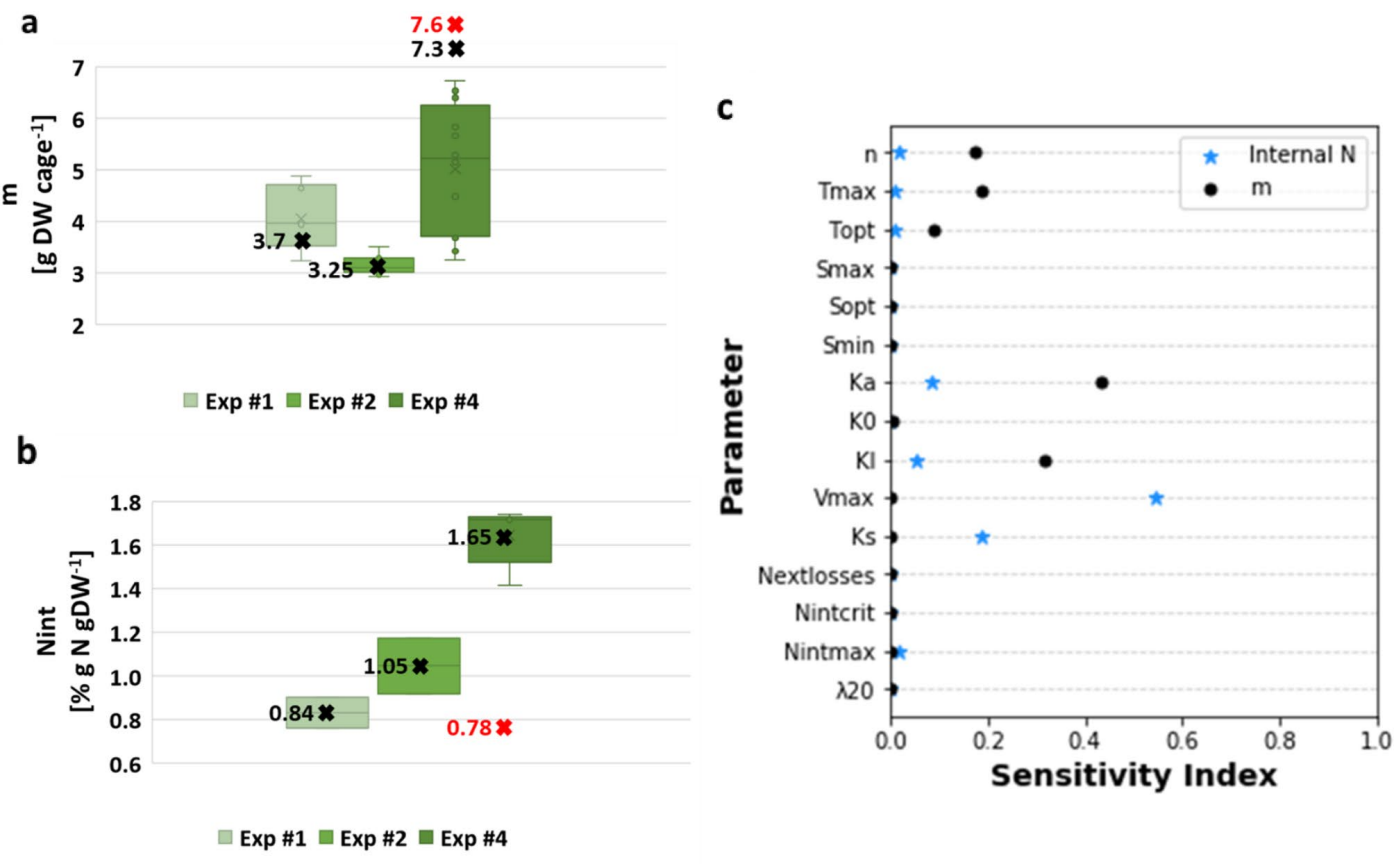
Modeled (marked by x) vs measured (in boxes) biomass **(a)** and internal N **(b)** of *Ulva* sp. cultivated for a period of 7–10 days in cages that were installed in a depth of 5 m cages offshore the EMS after continuous nutrient enrichment in experiments #1, #2 and #4. In experiment #4, the red x presents model predictions using a constant ${N}_{ext}$ of 0.75 µM N and the black x presents model predictions using a changing ${N}_{ext}$, as described in Table 5. **(c)** Illustrated sensitivity of simulated biomass production (black circles) and N content (blue stars) to model parameters, as measured by the Sobol method, in the offshore cultivation system.

Simulation results leading to the estimate of ${N}_{ext}$, are presented in Table 5, Fig. 3a, b and in Figs. S3–S5 in Suppl. Appendix C. In experiments #1 and #2, the simulation predicts the outcome of the DGR and internal N with satisfactory accuracy (RMSRE = 27.1 or 18.1%). In experiment #4 we observe a larger error (RMSRE = 45.1%) that we attribute to the incorrect settings of ${N}_{ext}$. Artificially adding external N at the time instant after the high wave event experienced on May 6, when the current direction changed, is helpful from the simulation point of view and improves the obtained result (RMSRE = 42.8%). Other modifications, e.g. irradiance or temperature cannot explain the results at the same efficiency.

We take the simulation results with caution. The accuracy of this model is lower but comparable to the accuracy of the controlled photobioreactor model (RMSRE of 15–21%)^43^ but high in comparison to literature models that predict *Ulva* sp. growth in natural environments (35–110%)^29^.

The sensitivity analysis of the model shows that it is sensitive to ${K}_{a}$ and ${K}_{I}$ (0.3–0.5, Fig. 3c). We could suggest that large biomass density in these unmixed cages leads to smaller light penetration depth and as a result, increased internal N storage. This does not diminish the dependence of internal N model on ${K}_{s}$ and ${V}_{max}$ (0.2–0.55), both pointing to dominant effect of the external N concentration in the environment. Biomass production in DGR is more sensitive to temperature-related parameters, ${T}_{opt}$, ${T}_{max}$ and *n* (0.1–0.2), such that temperature rise to around 30 °C during the summer can be set as a limiting, or maximum possible growth temperature for *Ulva* sp.^44^. Future studies could focus on additional effects of light absorption and active mixing of *Ulva* sp. biomass in offshore cages^11^.

In conclusion, we presented how with some minor adjustments, the model developed for a controlled mixed reactor can simulate the dynamics of biomass production and internal N in an offshore environment and a flat cage cultivation system. Furthermore, we developed a method to estimate N level in the sea during the cultivation period. This method should be validated by actual measurements of N concentrations (nitrate and ammonium) in the sea during an offshore experiment.

## Discussion

This study provided data to gain new insights on the effects of seasonality, depth, and pre-cultivation fertilization duration on growth rates and chemical composition in open-water offshore *Ulva* sp. biomass production. The data was used to develop a model which allows for the optimization of cultivation cycles and pre-cultivation fertilization requirements offshore.

We show that cultivation depth offshore is an important parameter affecting *Ulva* growth. The 5 m depth is more favorable for *Ulva* sp. cultivation than the 1m depth. The inferiority of the 1m depth in terms of lower growth rates (Fig. 2c) is strongly associated with the mechanical stress caused by the surface waves. This result is consistent with the approach of growing seaweed on submerged substrates at a typical depth of 3–10 m beneath the bulk of wave turbulence, which is a standard in exposed or offshore seaweed farming cultivation systems^15^.

Pre-cultivation fertilization significantly affects growth rates, mainly because the existing external N concentration in the EMS is insufficient for sustained growth. The high peak, short-term (20–24 h) fertilization treatment was ineffective in the examined conditions. This is in contradiction to previous results that measured a significant recovery of up to 0.95% g N g^−1^ DW in less than 12 h^45^. The idea of onshore preparation of the seaweed before offshore cultivation is not new^46^. Traditionally, seaweed spores and seedlings are maintained, propagated, and seeded on the cultivation rope/net and sometimes even fertilized^46^ in an onshore nursery, but a major part of the growth occurs in the sea, based on near/offshore nutrient concentrations. In an oligotrophic environment such as the EMS, however, growth will usually be limited by nutrient supply. Thus, onshore fertilizing and the initial N level at the beginning of the offshore cultivation period is essential.

The lowest growth rates offshore *Ulva* sp. were measured in the winter (December) and maximal growth rates in spring (May) and summer (July) (Fig. 2e). Although growth is possible during winter, the accumulated growth rates are low due to biomass losses during winter storms, turbidity, and strong currents. Such biomass losses due to storm action are a known yet toleratable phenomena, as long as the the long-term integrity of cultivation structures can be ensured^15^.

We developed a framework for using cultivation models to optimize the operation of this two-stage cultivation method. An offshore cultivation model can predict growth and decide upon harvest timing. An onshore pond/reactor cultivation model^43^ can be used to design the onshore fertilizing stage according to the required biomass and level of internal N for the offshore stage. In addition, the onshore stage can be used to fine-tune model parameters in different seasons, locations, or seaweed species for improved on and offshore model performance. Although harder to calibrate, the offshore model can develop into an important tool for governments and seaweed farmers, providing seasonal predictions that will enable better preparation and operation in an era of increasing climatic uncertainty (i.e., predict how sea water temperature rise will affect the yields).

In experiments #2 (November 2019) and #4 (May 2020), a net N uptake was measured, implying that environmental N levels were not as low as previously reported^10^. Unfortunately, due to technical limitations, ammonia quantification in water samples was performed only during the preliminary experiments and experiments #1–#2, constraining our ability to support this also with water analysis data. Nevertheless, ammonia measurements demonstrated values in the range of 0–1.5 µM NH_4_ and did not show a consistent seasonal trend. These values fit the models ${N}_{ext}$ estimations of 0.75–1.25 µM but are a bit lower than the 2 µM NH_4_ background values measured at a nearby site during the summer and autumn of 2012 in a study by Korzen et al.^10^.

The cultivation model explains the results, demonstrating that offshore N levels changed between experiments. The influx with the highest ${N}_{ext}$ concentration was estimated in May 2020. In two offshore cultivation experiments, from November 2019 and May 2020, extra N uptake was identified compared to expected uptake according to background ${N}_{ext}$ concentrations and model predictions. This extra N uptake could be associated with nitrogen effluents from the nearby Lev-Yam fish cages or anthropogenic nutrients discharged from the Alexander estuary. Based on an assessment of dynamic current regimes (Suppl Appendix C) and the fact that fish loads in the nearby fish cages were constant during the different experiments (personal communication), we estimate that the source of these elevated N levels is coastal, namely from the Alexander estuary.

This work sheds light on the seasonal growth dynamics and major environmental effects on *Ulva* sp. cultivation offshore the EMS. Our work emphasizes the importance of sufficient nutrient supply and the significant effect of surface water motion on offshore biomass yields. Notwithstanding, the presented analysis is limited in the scale and duration of the offshore experiments.

Another limitation is data scarcity, relating specifically to measurements of external N, internal N and biomass weight along the cultivation experiments. Notably, offshore nutrient and biomass data collection is expensive and not always technically feasible in commercial farms, and model estimations can help fill this gap. For example, by incorporating individual measurements of biomass, internal N and external N into the model, approximate levels of biomass, internal N and external N along the whole cultivation period can be simulated. Additional limitations relate to unknown variations in biomass condition and age between experiments, potentially affecting growth rates and sporulation events^47^, the lack of in-situ hydrodynamical data and the potential effect of phosphoros limitation. Furthermore, the proposed model, despite its potential contribution to the progress of the field, will require additional data to improve its predictive abilities. In addition, the usability of the model for farm management and farm scale prediction may be elevated by incorporating it into a multi-scale model^40^. Future studies need to test the performance of other cultivation systems under similar conditions and examine alternative fertilization methods. In addition, the profitability of fertilized offshore seaweed farming should be evaluated, specifically examining if the income from increased growth rates compensate for the added costs of fertilizing. The model should be further improved by relating to P limitation, water motion, and waves, and validated on more offshore data from experimental and commercial farms.

Finally, the study allowed determining the impact of location, cultivation depth, nutrient availability, technology, and seasonality on the *Ulva* species biomass yield and its chemical composition relevant for commodity production. To progress the economically viable macroalgae-based supply chains, the follow-up study should translate the described technology into an analytical production function and investigate conditions for profitable production from a private and public perspective. The private perspective is based on market potential, while the public perspective considers the monetized external costs and benefits that might follow from macroalgae utilization.

## Conclusions

We grew *Ulva* sp. offshore the EMS with a pre-cultivation onshore nutrient supply and developed a better understanding of seasonal growth dynamics and environmental effects (nitrogen, waves, depth, etc.). Specifically, we found that *Ulva* sp. is better cultivated deeper due to surface waves (i.e., 5m depth). In the EMS, sufficient nutrients should be provided before offshore cultivation. We developed a predictive model and validated it with data from our offshore experiments. This model has the potential to be used throughout the whole lifecycle of seaweed cultivation: from early nursery and farm design through economic models and estimations of environmental effects required by authorities and industry, ending with optimization of an ongoing farm operation.

## Methods

### Experimental part

#### Marine macroalgae biomass

Starting at the autumn of 2018, *Ulva* sp. biomass maintained in Tel Aviv University system^48^ was cultivated in a MPBR built on a southern wall in the aquaculture center in Michmoret (Fig. 1a). Nutrients were provided by constant exchange of nutrient rich seawater, pumped from the Michmoret bay. Throughout all experiments, the fresh weight (F.W.) of the biomass was determined using analytical scales after removing surface water using an electric centrifuge (Spin Dryer, CE-88, Beswin). Growth rates were calculated as Daily Growth Rate (DGR, Eq. (1), as recommended by^49^.

1 $$DGR=100\% \left({\left({{F.W.}_{out}}/ {{F.W.}_{in}}\right)}^\frac{1}{t}-1 \right)$$

where FW_i_ (g) is the initial fresh weight, FW_out_ (g) is the final fresh weight and t is the number of cultivation days.

#### Offshore cultivation systems 

Cultivation experiments of *Ulva* sp. offshore Michmoret enabled us to measure year-round growth rates and chemical compositions in the Israeli EMS and to test the performance of a cultivation model for naturally varying environmental offshore conditions. The EMS offshore environment is ultra-oligotrophic, which also enabled assessing the effectiveness of short-term onshore fertilization as a method for recharging depleted nutrient storages in biomass between offshore cultivation periods.

Two ring-shaped offshore macroalgae cultivation systems (Fig. 1e, f) were installed during November 2018 on a marking buoy of the Lev-Yam fish cages (Fig. 1b) located 3.2 km offshore Michmoret (Fig. 1d). The systems were welded from two flat 5mm*50mm stainless steel 316L rods bent into a large external ring (r = 0.95m), a small internal ring (r = 0.25m), and an internal connecting partial cross (Fig. 1e). Each system was installed on the buoy by connecting both parts with stainless steel screws underwater. The systems were installed in two depths: 1m and 5m (Fig. 1b). Each system was divided into four quarters and can carry up to twelve cultivation cages simultaneously (Fig. 1e). The cages (0.15 m × 0.3 m, total illuminated area 0.045 m^2^) were built from polyethylene (D = 32 mm), high-density polyethylene (HDPE) (D = 16 mm) pipes, and two layers of polypropylene tubular nets (TENAX, Gallo Plastik, Italy) to allow full illumination and prevent grazing. The external nets were green 74N140 nets with a mesh size of 12–14. The internal nets were white 32G223 and 40G223 nets with mesh sizes of 15–20. They were fortified with a second layer of polyethylene net with smaller mesh holes to minimize biomass loss due to thalli fragmentation.

#### Offshore cultivation experimental setup

Four successful experiments were performed between July 2019 and April 2020 (Table 6). A preliminary experiment was performed at the end of May 2019. In this experiment, the growth rates measured in the first cultivation period (after continuous fertilization) were invalid due to significant biomass losses due to large holes in the net. However, the growth rates measured in the second cultivation period were valid as we fortified the internal net between the first and the second cultivation periods.

At the beginning of each experiment, cages were stocked onshore with 20g F.W. of freshly harvested *Ulva* biomass from the Michmoret MPBR (Fig. 1a), stitched tightly, transported by boat to the site and installed on the system by scuba divers (Fig. 1b). The time the biomass was outside water was kept to an unavoidable minimum. On the harvesting day, the cages were collected by scuba divers and returned to shore, where all biomass was taken out of the cages manually for weighing and storage for further chemical analysis. Finally, cages were cleaned thoroughly and stored for the next experiment.

As described in Table 6, different experiments emphasize various aspects of offshore cultivation. In the preliminary and the first experiments, we examined whether a day of fertilization after a week of cultivation offshore the EMS (starvation conditions) supports further growth. This was investigated by performing two consecutive offshore cultivation periods separated by 18–24 h of onshore fertilization. In the third experiment, performed during the winter, we examined if more extended cultivation periods could be applied without compromising the daily yield. In this experiment, six cages were collected after seven days and six were collected after 12 days. The rationale of this design was that during the winter, there are fewer daily hours of light, and more extended cultivation periods may be needed to fulfill the biomass production potential.

**Table 6:** Details of offshore cultivation experiments.

	Cultivation period 1*	Depth (m)	Cages #	Depth (m)	Cages #	Cultivation period 2*	Depth (m)	Cages #	Depth (m)	Cages #
Preliminary experiment	20.5.19–28.5.19	1	1–6	5	13–18	29.5.19–6.6.19	1	1, 3, 4, 6	5	13–18
Experiment 1	22–29.7.19		1–6		13–18	30.7.19–5.8.19		1–6		13–18
Experiment 2	30.10.19–6.11.19		1–9		13–21	–		–		–
Experiment 3	11.12.19–18.12.19		–		19–24	11.12.19–23.12.19**		–		13–18
Experiment 4	30.4.20–12.5.20		–		13–24	–		–		–

*Cultivation period 1 began after a long-term continuous fertilization period, whereas cultivation period 2 began after a short one-day fertilization period.

**In experiment 3, all cages were installed after continuous fertilization. The difference between period 1 and period 2 in this experiment is only cultivation duration.

#### Onshore fertilization

A dedicated onshore experiment examined the effectiveness of 24-h of fertilization of low nitrogen *Ulva* biomass from EMS offshore cultivation. The experiment tested if soaking low-N *Ulva* biomass after a week offshore, in a high-nutrient solution for 18–24 h can recharge enough N to enable an additional week of growth.

The fertilization experiment was performed in two repetitions, during the preliminary cultivation experiment and experiment 1. Fertilization was applied in outdoor aerated 35L tanks, filled with nutrient-rich seawater. Half of the tanks were enriched in additional 1000 µM NH_4_ and 200 µM PO_4_ (Fig. 1c). Cages were collected from the offshore system on the morning (9:00/11:00 of the 28.5.2019/29.7.2019). Fertilizing started at 13:00/16:00 after weighing, harvesting and restocking, and continued for 18–24 h. The weight of the restocked biomass varied according to the amount of losses during the first cultivation period.

After fertilization, the cages were returned offshore for an additional cultivation period. Water was sampled in the fertilization tanks at the end of each fertilization period. Finally, the fertilization effectiveness was inferred from the growth rate and chemical composition measured in the consecutive offshore cultivation period.

#### Water sampling

Water sampling was performed in plastic syringes, filtered through a 0.2 µm filter to prevent particulate and microbial contamination, and kept at −20 ˚C until analysis. Offshore water was sampled in every installation or collection of cages, at a 5 m depth (by scuba divers) and at the sea surface (from the boat), representing 1 m depth. Water was also sampled during the fertilization experiments, as described above.

#### Temperature and irradiance

Temperature and irradiance were measured in each offshore experiment by a Onset^®^ HOBO^®^ sensor UA-002-08 (Onset Inc. MA), placed in one representative cage in each depth. The device also collected data during the onshore fertilizing period. In addition, as a backup for cases in which the HOBO devices were damaged or lost in the sea (Table S4), we used two more sources: (1) irradiance data from all experiment periods was extracted from the IMS data base from the Israel Meteorological Services (https://ims.data.gov.il/ims/1), and (2) water temperature was extracted from the Israel Marine Data Center (ISRAMAR) station located at a depth of 12 m, 2.3 km offshore Hadera and 8 km North of the cultivation site in Michmoret. The suitability of the ISRAMAR data for the offshore model was determined based on a comparison to the available temperature measurements from the HOBO devices.

#### Biomass chemical composition analysis

At the end of each cultivation experiment, biomass samples were harvested, weighed (F.W.), dried in 40–60 ˚C, grinded with a mortar and pestle (and using liquid nitrogen if needed), and then kept at 4 ˚C until further analysis. ~ 70% of the samples underwent elemental analysis.

#### Elemental analysis

Elemental analysis for C, H, N, and S content as % of D.W. was performed at the Technion, Chemical, and Surface Analysis Laboratory, using Thermo Scientific CHNS Analyzer (Flash2000).

#### Water analysis (ammonia determination)

Ammonia concentration in water samples was determined following the method of Holms *et* al. (1999)^50^. Water samples were diluted using ultra-pure water aiming for concentrations lower than 0.5 µM NH_4_, which are optimal for this method.

#### Data analysis

##### Fertilizing treatment

The effect of continuous and rapid fertilization on growth rates and internal N were compared using the two-tailed Mann–Whitney U test (groups # = 2, DF = 1).

##### Cultivation depth

The effect of cultivation depth, specifically 1m and 5m, on growth rates and internal N, were compared using the two-tailed Mann–Whitney U test (groups # = 2, DF = 1).

##### Cultivation date

Growth rates and internal N in different dates were compared using the Kruskal–Wallis H test (groups # > 2, DF > 1), followed by the post-hoc Dunn’s test with the Bonferroni adjustment method for pairwise comparison.

##### Cultivation duration

The effect of cultivation duration, specifically 7 and 12 days, on growth rates and internal N, was assessed using the two-tailed Mann–Whitney U test (groups # = 2, DF = 1).

##### Correlation between DGR and internal N

A two-tailed Pearson test was used to compare and test the correlation between DGR and internal N results.

##### Analysis tools

Statistical analysis was performed using Python (3.7.3), specifically the scipy folder (1.4.1).

### Model

Our model is based on the *Ulva* sp. dynamic cultivation model developed by^40^. The model focuses on reactor scale *Ulva* sp. cultivation in offshore conditions and was constructed to study environmental effects on internal N and biomass growth dynamics in the offshore environment.

#### Model assumptions

The model follows the basic assumptions of the original model, developed by Zollmann et al. (2021) in^40^, specifically that the dynamics of biomass growth and chemical composition are predicated by the dynamics of the limiting nutrient, in the case of the EMS nitrogen (N), under the constraining effect of light intensity (*I*). In the modeled environment, light intensity and temperature vary daily and seasonal, but salinity is relatively constant and was assumed to be 39 PSU. Therefore, the salinity (S) function was removed from the model (fS = constant), as constant S does not affect the system's dynamics.

Following the concepts of the Droop equation^51^, we assume that the effect of the concentration of the external N in the sea (${N}_{ext}$) on growth rate is not direct, but is mediated by the internal N in the biomass^23,52^. On the other direction, we assume that the biomass does not affect ${N}_{ext}$, an assumption which will need to be reexamined in larger scales.

Our model also assumes that the organic carbon reserve, accumulated during the photosynthesis process, is not limiting within the modelled conditions and that all growth occurs during the light-period.

#### Model governing equations

The model is based on two governing ordinary differential equations (ODEs), describing the mass balance of two state variables: biomass density in the cage (m, g Dry Weight (D.W.) L^−1^, Eq. 2) and biomass internal concentration of N (${N}_{\mathrm{int}}, \%\mathrm{ g N }{\mathrm{g DW}}^{-1}$, Eq. 7), under constant ${N}_{ext}$ and salinity. Both ODEs were solved numerically with hourly time steps.

2 $$\begin{aligned} &\frac{\partial m}{\partial t}= \left(\widehat{\upmu }-\widehat{\lambda }\right)m,\\ & \widehat{\upmu }={\widehat{\upmu }}_{max}f,f=\mathrm{min}\{{f}_{{N}_{int}},{f}_{I},{f}_{T}\} \\ &{Initial Condition (I.C): m}_{(t=0)}={m}_{0} \end{aligned}$$

Where $\widehat{\upmu }$ is the growth rate function in the offshore cage, ${\widehat{\upmu }}_{max}$ (h^−1^) is the maximum specific growth rate under the applied salinity (39 PSU) conditions, and $f$ is the combined growth function, made of ${f}_{{N}_{int}}$ (Eq. 5) and ${f}_{I}$ (Eq. 6), which are the ${N}_{\mathrm{int}},$ and *I* growth functions. $\widehat{\lambda }$ (Eq. 3) is the biomass specific losses rate as at S = 39 $PSU$. $\widehat{\lambda }$ does not relate to losses in sporulation events. As described in^40^, all rates appear on a per hour basis.

3 $$\widehat{\lambda }={\widehat{\lambda }}_{20} {\theta }^{T-20}$$

where ${\widehat{\lambda }}_{20}$ (h^−1^) is the specific rate of biomass losses and $\theta$ is an empiric factor of biomass losses.

4 $${f}_{Temp}=\mathrm{exp}\left(-2.3{\left(\frac{T-{T}_{opt}}{{T}_{x}-{T}_{opt}}\right)}^{n}\right)$$

where ${T}_{x}={T}_{min}$ for $T\le {T}_{opt}$ and ${T}_{x}={T}_{max}$ for $T>{T}_{opt}$. ${T}_{min}$, ${T}_{opt}$ and ${T}_{max}$ ($^\circ{\rm C}$) are the minimal, optimal and maximal temperatures for *Ulva* growth.

5 $$\begin{aligned} & {f}_{{N}_{int}}=\frac{{N}_{\mathrm{int}}-{N}_{\mathrm{int min}}}{{N}_{\mathrm{int}}}/ \frac{{N}_{\mathrm{int crit}}-{N}_{\mathrm{int min}}}{{N}_{\mathrm{int }crit}} \mathrm{ for } \quad {N}_{int}<{N}_{\mathrm{int crit}}, \\ & \mathrm{or } \quad { f}_{{N}_{int}}=1 \quad \mathrm{ for } \,{N}_{int}>{N}_{\mathrm{int crit}} \end{aligned}$$

where ${N}_{\mathrm{int min}}$ and ${N}_{\mathrm{int ma}x}$ (% g N g DW^−1^) are the minimum and maximum internal N concentrations in *Ulva*, respectively, ${N}_{crit}$ (% g N g DW^−1^) is the threshold ${N}_{\mathrm{int}}$ level below which the growth rate slows down.

${f}_{I}$ (Eq. 6) was adjusted from the original light-function (Eq. 6 in^40^) as: (1) The z dimension of a flat cage is very small, and (2) the system was located 5 m under the sea surface, requiring an additional light absorption term. Therefore, we added light extinction in the water column and assumed that light extinction in the water inside the cage is negligible.

$$f\left(I\right)=\frac{{I}_{average}}{{K}_{I}+{I}_{average}}PAR$$

6 $${I}_{average}=\frac{({I}_{0}-{K}_{0}{Z}_{wc})}{{K}_{a}SD}\left[1-\mathit{exp}\left(-\left({K}_{a}SD\right)\right)\right]$$

where ${I}_{average}$ and ${I}_{0}$ (μmol photons m^−2^ s^−1^) are average photon irradiance in the cage and incident photon irradiance at the water surface, respectively, ${K}_{I}$ (μmol photons m^−2^ s^−1^) is the light half-saturation constant that is also affected by photosynthesis efficiency^53^ (a low ${K}_{I}$ is associated with a high photosynthesis efficiency and vice versa), $SD$ (g D.W. m^−2^) is stocking density of biomass per unit of water surface in the cage, ${K}_{0}$ (m^−1^) is the light extinction coefficient in the water, ${Z}_{wc}$ (m) is water column depth above the cage and ${K}_{a}$ (m^2^ g^−1^ DW) is the *Ulva* light extinction coefficient.

7 $$\begin{aligned} & \frac{\partial {N}_{int}}{\partial t}={\psi }_{{N}_{ext}}-{N}_{\mathrm{int}}f\mathrm{m}\\ & {\psi }_{{N}_{ext}}=\frac{{N}_{\mathrm{intmax}}-{N}_{\mathrm{int}}}{{N}_{\mathrm{intmax}}-{N}_{\mathrm{intmin}}}\frac{{V}_{\mathrm{max}}{N}_{\mathrm{ext}}}{{K}_{S}+ {N}_{\mathrm{ext}}}\\ & {\mathrm{I}.\mathrm{C}: {N}_{\mathrm{int}} }_{(t=0)}={{N}_{\mathrm{int}} }_{0} \end{aligned}$$

Where ${\psi }_{{N}_{ext}}$ (μmol-N gDW^−1^ h^−1^) is the N uptake function, formulated of ${N}_{\mathrm{intmax}}$ and ${N}_{\mathrm{intmin}}$ ($\%\mathrm{ g N }{\mathrm{g DW}}^{-1})$, ${V}_{\mathrm{max}}$ (μmol-N gDW^−1^ h^−1^), the maximum N uptake rate and ${K}_{\mathrm{S}}$ (μmol-N l^−1^), the N half-saturation uptake constant. $-{N}_{\mathrm{int}}f\mathrm{m}$ describes ${N}_{int}$ dilution in biomass by growth.

#### Model parameters

Model parameters (Table 4) were determined in a four-steps calibration process. First, ${\widehat{\upmu }}_{max}=0.03$ Light h^−1^ and ${N}_{int min}$= 0.48% g N g^−1^ DW were determined according to experimental results and ${T}_{min}$ was set as $4$ °C as described in^43^. Next, data from three different cultivation experiments was used to determine the remaining parameters, inside a predefined range. This was done using a Sequential Least SQuares Programming optimizer (SCIPY) to minimize the Root Mean Square Relative Error (RMSRE) between model predictions and experimental data.

In the second step, Parameters 4–10 ($\widehat{\lambda }$, ${N}_{int max}$, ${N}_{int crit}$, ${K}_{s}$, ${V}_{max}$, ${K}_{I}$ and ${K}_{0}$) were determined based on data from experiments of *Ulva* cultivation under various fertilizing regimes in a photobioreactor with controlled light and constant temperature and salinity^43^. In the third step, Parameters 11–16 (${S}_{min}$, ${S}_{opt}$, ${S}_{max}$, ${T}_{opt}$*,* ${T}_{max}$ and *n*) were determined based on data from experiments of *Ulva* cultivation in various mixes of nitrate-rich desalination brine and Artificial Seawater (ASW) (Suppl Appendix A). It should be noted that steps 2 and 3 of the calibration process were performed using the model with its original light function (Eq. 6 in^40^), before it was adjusted to the offshore system. Forth, parameter 17, ${K}_{\mathrm{a}}$, and the ${N}_{\mathrm{ext}}$ levels during each experiment were evaluated by minimizing the average RMSRE between *m* and ${N}_{\mathrm{int}}$ for each experiment (${N}_{\mathrm{ext}}$) and for all experiments together (${K}_{\mathrm{a}})$.

#### Model simulations

Due to a lack of data regarding the exact levels of ${N}_{ext}$, we estimated ${N}_{ext}$ for each cultivation experiment by running the model for multiple ${N}_{ext}$ levels (in the range of 0–8 μmol N L^−1^) and choosing the value with the smallest RMSRE between predictions and measurements. In some cases, in which predictions and measurements did not converge, we had to split the modeled cultivation period to a few shorter periods with different ${N}_{ext}$ levels. This was done while considering the changing current regime that could potentially lead to nutrient enrichment in specific days of the cultivation period. Finally, we simulated biomass and ${N}_{int}$ dynamics for each experiment, presenting predicted and measured levels of biomass and ${N}_{int}$ at the end of each experiment.

### Supplementary Information

Supplementary Information.

## Data Availability

All data generated or analysed during this study are included in the Data for "Cultivation of *Ulva* sp. offshore the Eastern Mediterranean Sea in experimental bioreactors: seasonal growth dynamics and environmental effects" repository^54^, osf.io/u2mhk.
