# Supplementary material for: Effects of season, depth and pre-cultivation fertilizing on Ulva growth dynamics offshore the Eastern Mediterranean Sea
Source: Sci Rep. 2023 Sep 7;13:14784. doi: 10.1038/s41598-023-41605-4 (PMC10485012; doi:10.1038/s41598-023-41605-4)
Supplement: Supplementary file 1 — Supplementary Information. [file 41598_2023_41605_MOESM1_ESM.docx]

**Supplementary materials for**

**Effects of season, depth and pre-cultivation fertilizing on *Ulva* growth dynamics offshore the Eastern Mediterranean Sea**

Submitted to

**Scientific Reports**

Meiron Zollmann^a,^* Alex Liberzon^b^, Ruslana R. Palatnik^c^, David Zilberman^d^ and Alexander Golberg^a^

^a^Porter School of Environmental and Earth Sciences, Tel Aviv University, Tel Aviv, Israel

^b^School of Mechanical Engineering, Tel Aviv University, Tel Aviv , Israel.

^c^Department of Economics and Management, The Max Stern Yezreel Valley College, Israel. NRERC- Natural Resource and Environmental Research Center, University of Haifa, Haifa, Israel

^d^Department of Agricultural and Resource Economics, The University of California at Berkley, Berkeley, CA, USA

*Corresponding author: Meiron Zollmann

Email: meironz@tauex.tau.ac.il

# Appendix A - outdoor semi-controlled bottles photobioreactor

**Introduction**

The outdoor semi-controlled bottles photobioreactor was established as part of study aimed to examine the effectiveness of *Ulva* sp. cultivation in nitrate rich ground water desalination brine for the sake of nitrate removal and biomass production. This study included an experimental part, in which *Ulva* was cultivated in various dilutions of brine and ASW and different stocking densities, measuring growth rates and internal N content at the beginning and the end of each experiment, and a modeling part, in which the reactor scale cultivation model was adjusted to this system.

**Methods**

**Cultivation system**

Twelve upside down 1.5L transparent PET bottles, positioned on the Southern wall of the Porter building in TAU, were used as photobioreactors (Figure S3). Each reactor was filled up with 1 L composed of a dilution of ASW (39 PSU) and nitrate rich ground water desalination brine (6 PSU, 265 mg- NO_3_^-^ L^-1^), provided from the Israeli water company, Mekorot, from the Ashkelon brine facility. The bottles were well mixed by bottom aeration and no water exchange was performed.

**
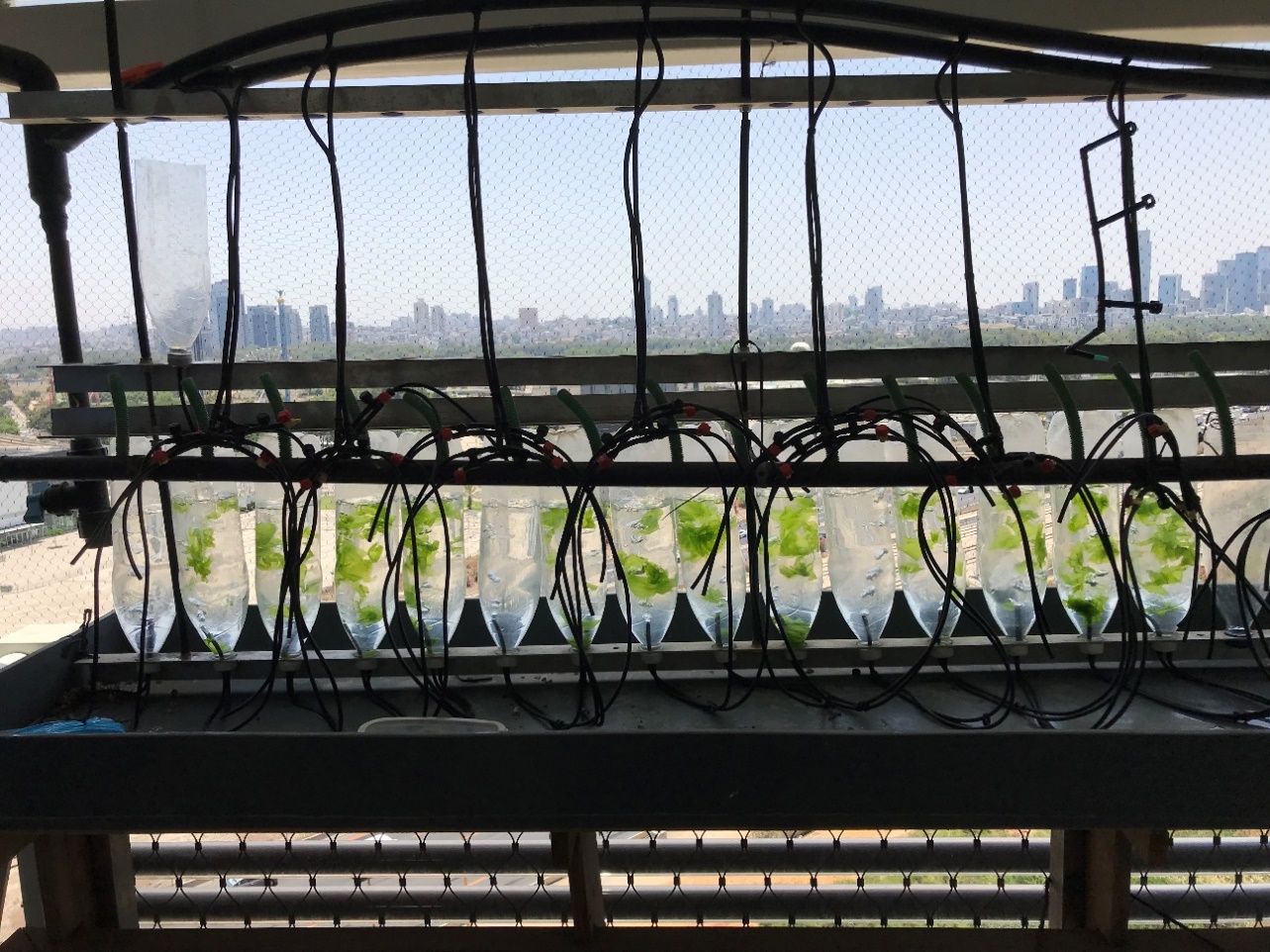
**

**Figure S1**. Outdoor bottles photobioreactors stocked with Ulva thalli during a cultivation experiment in various dilutions of nitrate rich ground water desalination brine and ASW.

**Experimental setup**

*Ulva* sp*.* was cultivated in the outdoor bottles photobioreactor*s* for two consecutive periods of 6-7 days during June 2020, examining the effects of four dilution ratios of ASW and nitrate rich brine and three stocking densities (1, 2 and 3 g F.W. l^-1^), as described in Table S6. Phosphorus was not supplemented to the cultivation reactors but is expected to be required for longer cultivation periods.

Experiment measurements focused on growth rate and N content (CHNS analysis), while the environmental parameters temperature and irradiance were monitored for control by a HOBO device that was positioned in an adjacent bottle, filled only with ASW.

**Table S1. Experimental data used for calibration of outdoor semi-controlled bottles photobioreactor**

| Comment | Final Nint  [% g N gDW] | Final m  [g FW L^-1^] | Stocking density [g FW L^-1^] | Initial nitrate concentration [mg-NO_3_ L^-1^]^*^ | Salinity  [PSU] | Dilution rate  [L brine : L ASW] | Bottle # | experiment # |
| --- | --- | --- | --- | --- | --- | --- | --- | --- |
|  | 3.1, 2.3 | 1.3, 2.1 | 1 | 265 | 6 | 1:0 | 2,3 | 1^**^ |
|  | 2.5, 2.8 | 4, 3 | 2 |  |  |  | 4,5 |  |
|  | 2.4, 2.1 | 3.8, 4.5 | 1 | 132.5 | 24 | 0.5:0.5 | 7,8 |  |
|  | 1.6, 1.6 | 7.8, 8.1 | 2 |  |  |  | 9,10 |  |
|  | 2, 1.9 | 3.7 ,3.5 | 1 | 88.3 | 29 | 0.33:0.67 | 12,13 |  |
|  | 1.4, 1.9 | 6.9, 5.3 | 2 |  |  |  | 14,15 |  |
|  | 2.9, 3.2 | 3, 2.6 | 2 | 265 | 6 | 1:0 | 2,3 | 2^***^ |
| Salted brine | 2.8, 2.6 | 5, 4.6 | 2 | 265 | 40 |  | 4,5 |  |
|  | N/A, 2.2 | 4.4, 5.7 | 2 | 132.5 | 22.5 | 0.5:0.5 | 7,8 |  |
|  | 2.9, 2.3 | 5.7, 5.8 | 3 |  |  |  | 9,10 |  |
|  | 2.9, 2.2 | 5.1, 4.7 | 2 | 176.7 | 17.5 | 0.67:0.33 | 12,13 |  |
|  | 2.8, 2.4 | 6.6,6.9 | 3 |  |  |  | 14,15 |  |
| ^*^All concentrations are calculated based on the Mekorot’s report on a concentration of 265 mg NO_3_ L^-1^, and a negligible concentration in the ASW | | | | | | | | |
| ^**^ Nint,0 = 3.03 % g N g^-1^ DW | | | | | | | | |
| ^**^ Nint,0 = 4.09 % g N g^-1^ DW | | | | | | | | |

# Appendix B - Daily growth rates and internal N after rapid and continuous nutrient enrichment in depths of 1 m and 5 m

Figures S4 compares the results of each depth (1 and 5 meters cages) separately (p-value < 0.001). This figure shows a consistent but relatively mild effect on internal N in both depths.

**
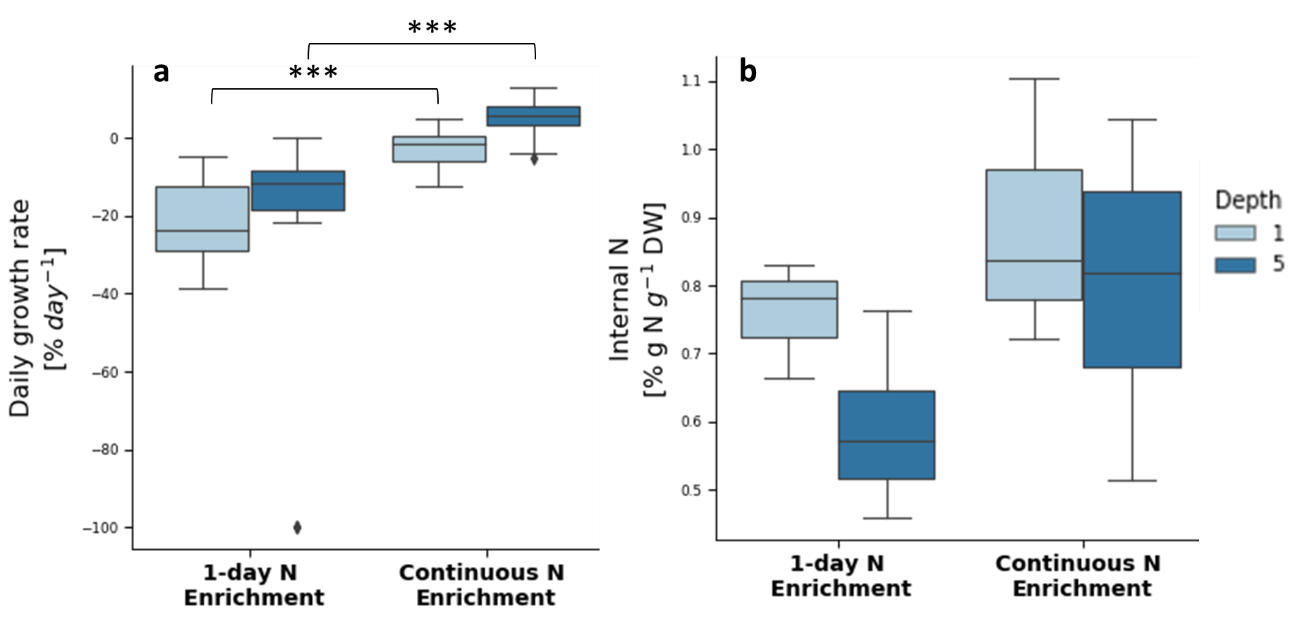
**

**Figure S2**. Daily growth rates **(a)** and internal N **(b)** of *Ulva* sp. cultivated offshore the EMS after rapid nutrient enrichment and after continuous nutrient enrichment in depths of 1 m **(light blue)** and 5 m **(dark blue)**, in the preliminary and in experiment #1**.** Asterisks indicate statistical significance of difference with *** p < 0.001, calculated by the two-tailed Mann-Whitney U test. # samples: rapid nutrient enrichment: 6 for DGR and 2 for internal N for each depth. continuous nutrient enrichment: 15 and 39 for DGR and 6 and 8 for internal N, for depth of 1 and 5 m, respectively.

# Appendix C - Offshore weather conditions during cultivation experiments

**Introduction**

*Ulva* cultivation offshore is highly dependent on meteorological conditions such as light intensity, water temperature, waves, currents, and winds. The goal of this Appendix is to provide graphic illustrations of those conditions, when relevant, to help and interpret the effect of those time dependent factors on results of the offshore *Ulva* sp. cultivation experiments.

**Methods**

This Appendix incorporates data from the IMS data base from the Israel Meteorological Services (<https://ims.data.gov.il/he/ims/6>) and from the IOLR ISRAMAR and Mediterranean GLOSS #80 station, located 2.3 km offshore Hadera. All data is plotted in per experiment plots.

**Results**

Light intensity and water temperature measurements along the offshore cultivation periods are presented among models simulations for experiments #1-2 and #4 (Figure S3-S5) and herein in Figure S6. This data is used to interpret the results of the offshore cultivation experiments, and as an input to the offshore cultivation model.


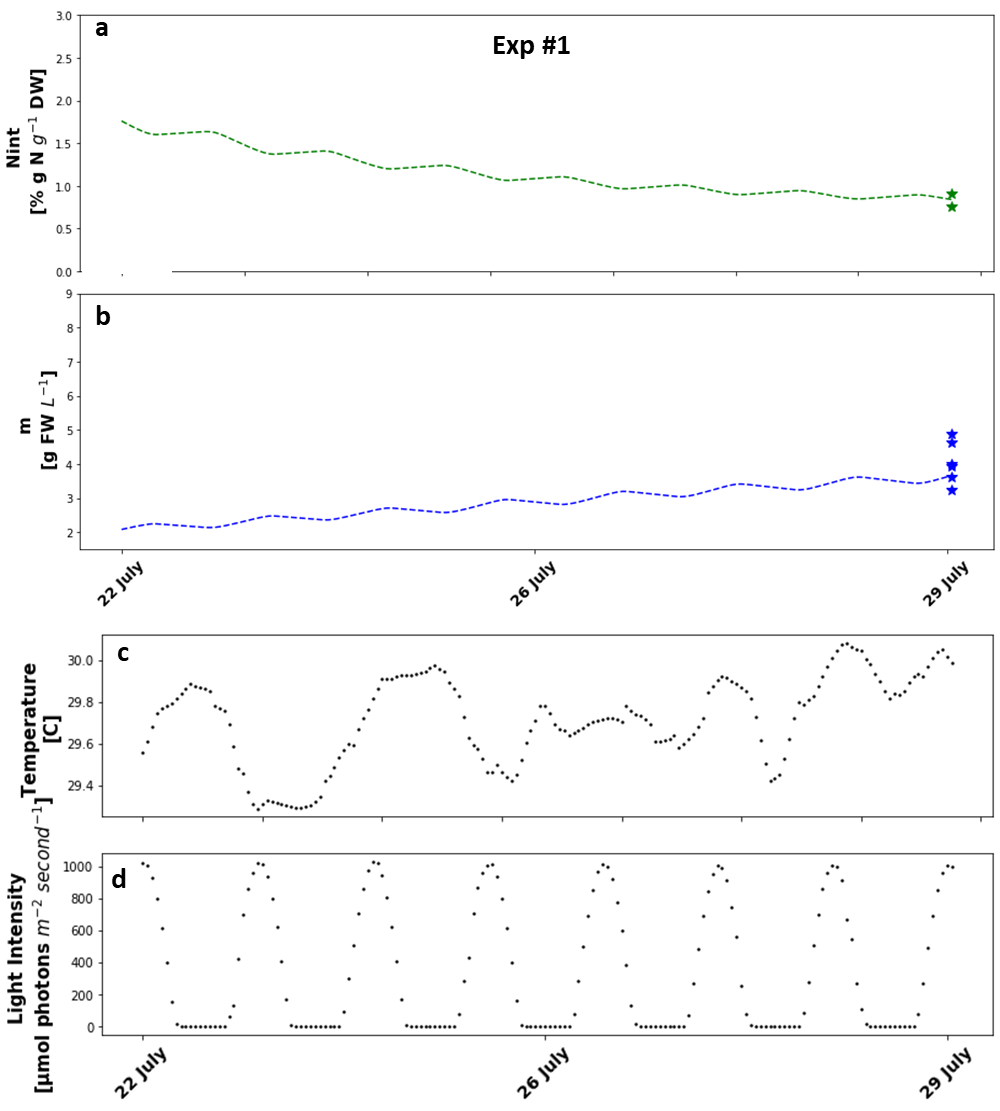


**Figure S3**. Model timewise simulation of *Ulva* sp. cultivation in offshore cages for a period of 7 days in experiment #1. Two variables are followed: $N_{int}$ (% g N g^-1^ DW, **a**) and m (g DW L^-1^, **b**). Biomass initial conditions: 2.08 g D.W. per L^-1^ (20 g F.W. per cage). $N_{int}$ initial conditions: 1.76% g N g^-1^ D.W. $N_{ext}$ initial conditions: 0.75 µM N. Empiric data points are presented in stars. (**c**) and (**d**) Temperature and light intensity profiles.

~~
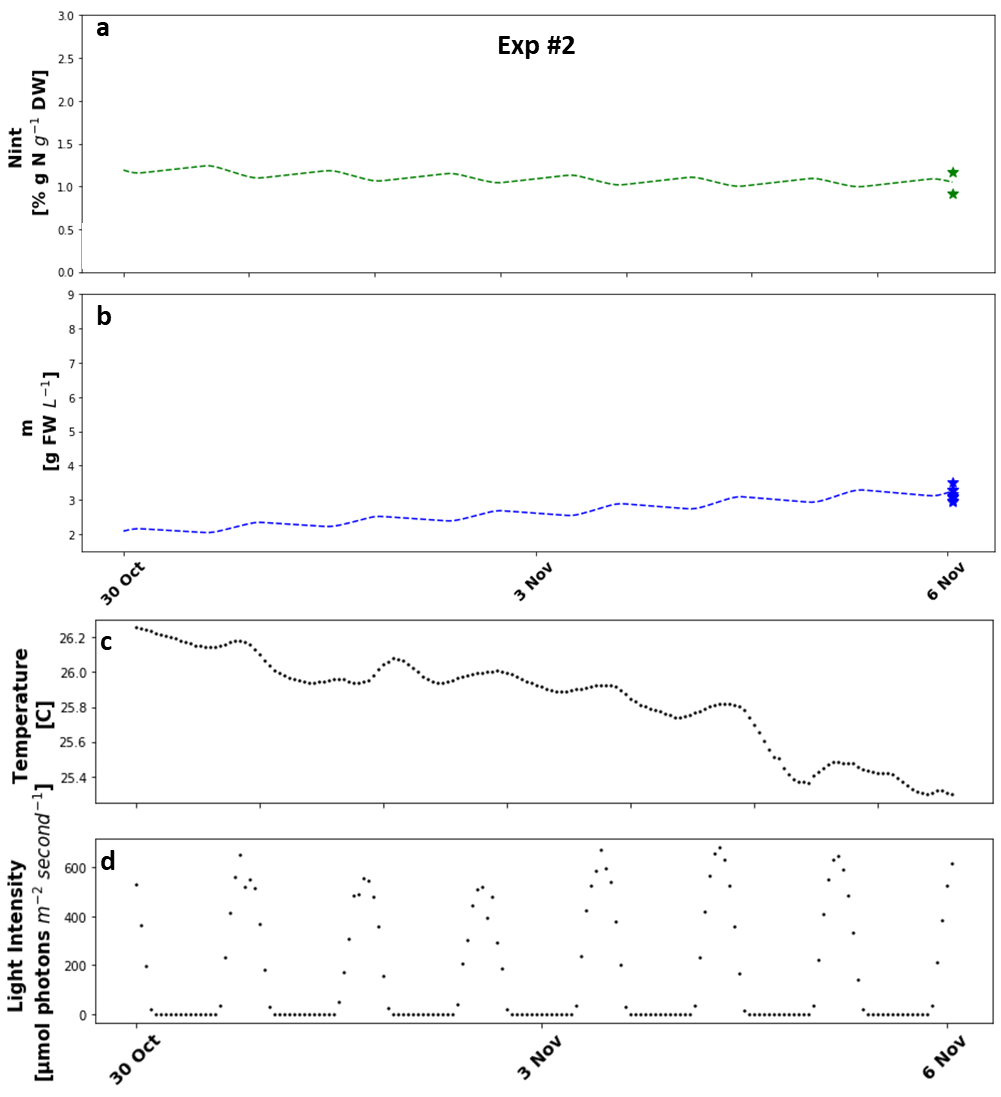
~~

**Figure S4**. Model timewise simulation of *Ulva* sp. cultivation in offshore cages for a period of 7 days in experiment #2. Two variables are followed: $N_{int}$ (% g N g^-1^ DW, **a**) and m (g DW L^-1^, **b**). Biomass initial conditions: 2.08 g D.W. per L^-1^ (20 g F.W. per cage). $N_{int}$ initial conditions: 1.19 % g N g^-1^ D.W.. $N_{ext}$ initial conditions: 1.25 µM N. Empiric data points are presented in stars. (**c**) and (**d**) Temperature and light intensity profiles.


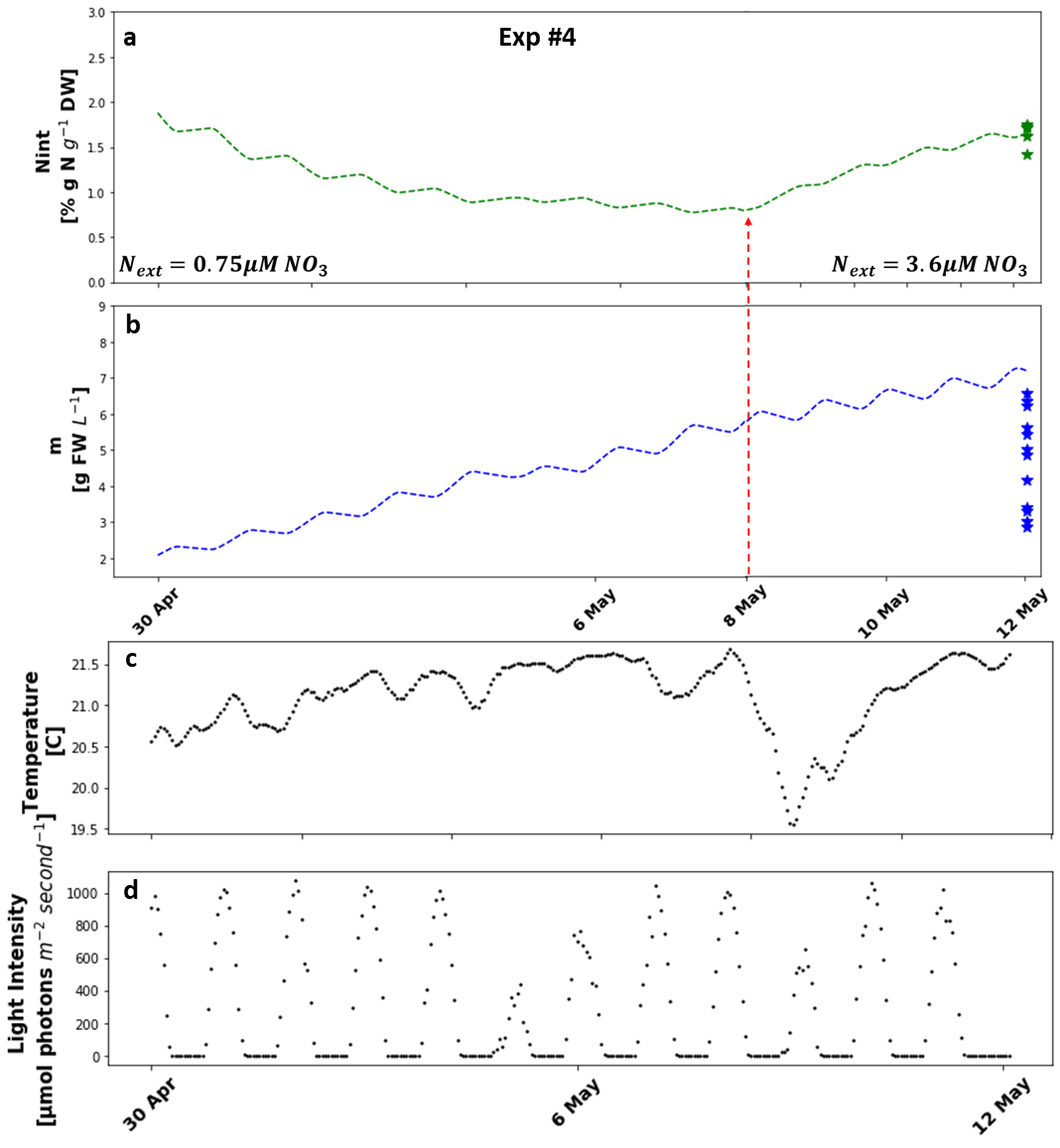


**Figure S5**. Model timewise simulation of *Ulva* sp. cultivation in offshore cages for a period of 12 days in experiment #4. Two variables are followed: $N_{int}$ (% g N g^-1^ DW, **a**) and m (g DW L^-1^, **b**). Biomass initial conditions: 2.08 g D.W. per L^-1^ (20 g F.W. per cage). $N_{int}$ initial conditions: 1.88 % g N g^-1^ D.W. $N_{ext}$concentration in seawater changes from 0.75 µM N initially to ~3.6 µM N after the May 8 (marked by red arrow). Empiric data points are presented in stars. (**c**) and (**d**) Temperature and light intensity profiles.


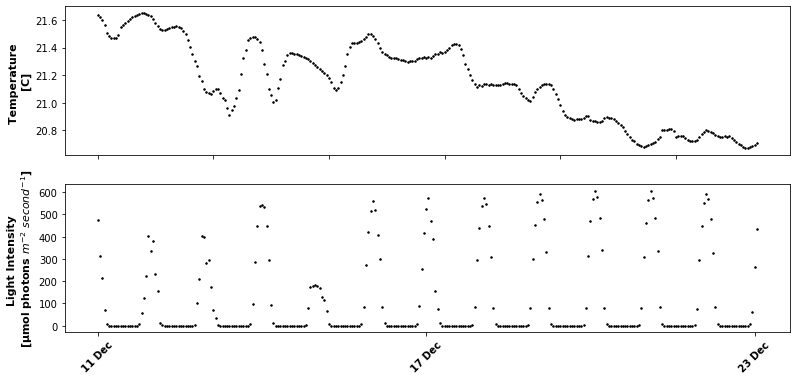


**Figure S6.** Water temperature **(top row)** and light intensity **(bottom row)** measured during experiment #3.

Significant wave heights (height of upper third of the waves) along the offshore cultivation periods are presented in Figure S7. Focusing on the periods we the higher waves (above 2 meters), we present in Figure S8 the wind directions during the days before the rising of the waves in experiments #3 (11.12.2019) and #4 (4-5.5.2020). The figures show that on the 11.12.2019, the dominant wind direction was easterly (western wind), whereas on the 4-5.5.2020 the dominant wind direction was westerly (eastern wind).


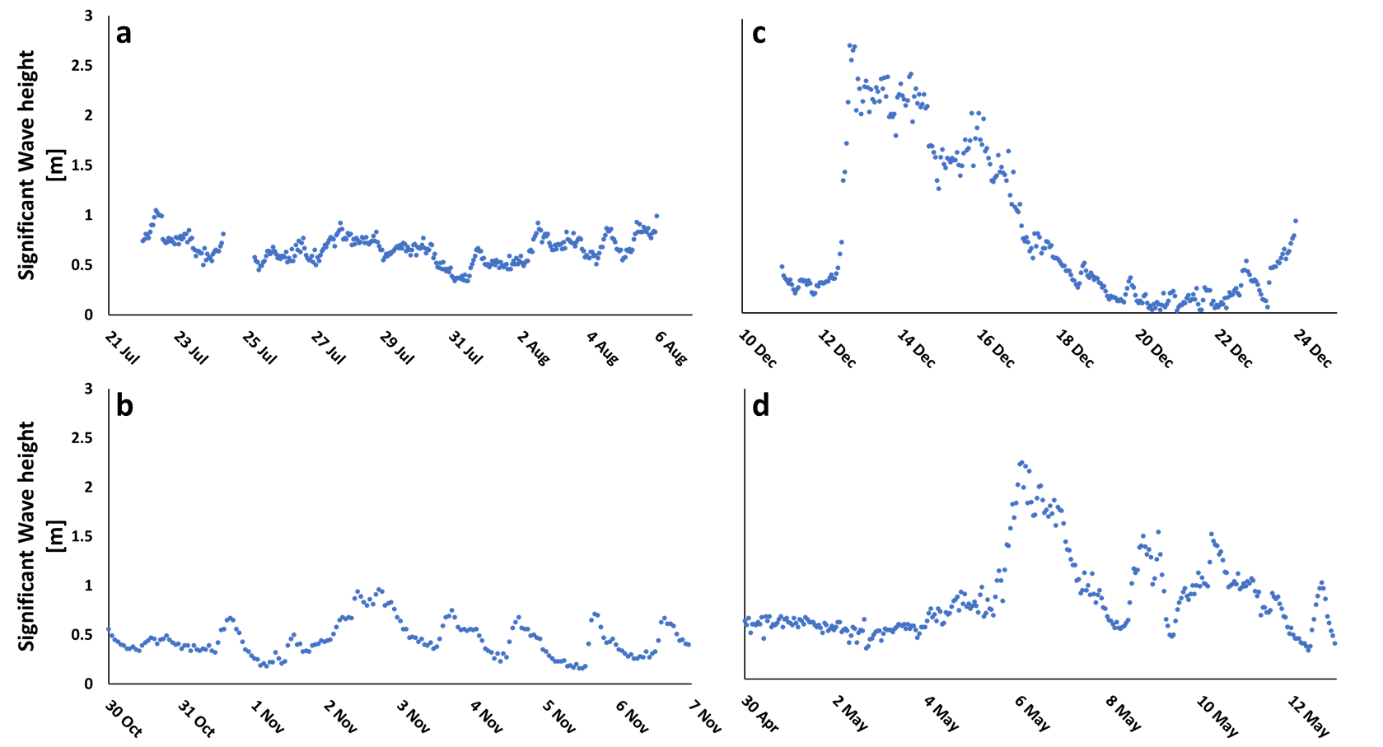


**Figure S7**. Significant wave heights during experiments #1 **(a)**, #2 **(b)**, #3 **(c)** and #4 **(d)** of the offshore Ulva sp. cultivation experiment, as measured in the Hadera GLOSS #80 station. Experiments #1-#3 were performed in 2019 and experiment #4 was performed in 2020.


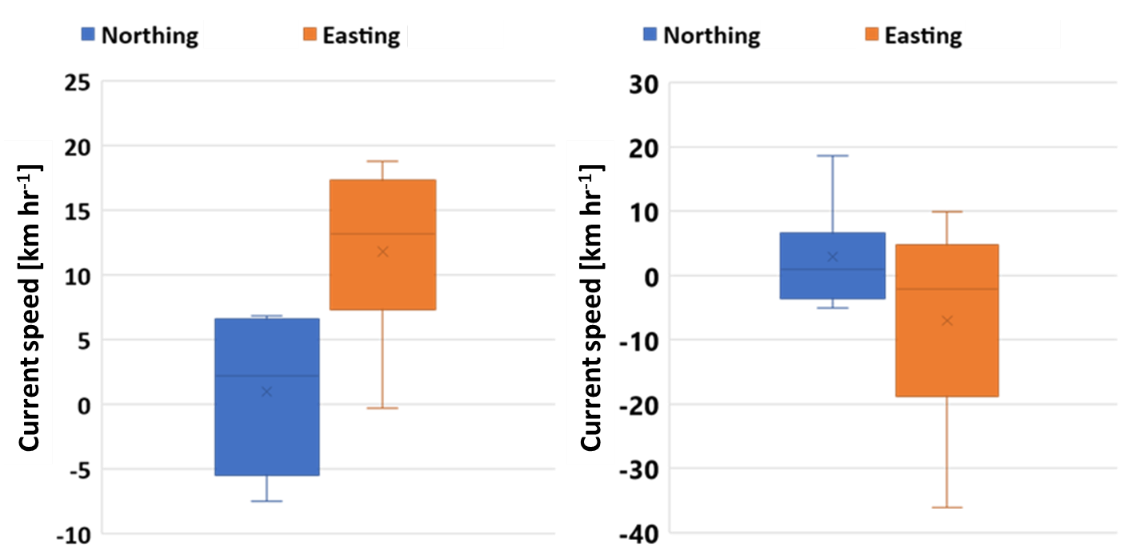


**Figure S8.** Wind direction and speed before the rising of the waves in experiment #3, during the 11.12.2019 **(left)**, and in experiment #4, during the 4-5.5.2020 **(right)**.

Current regime during the cultivation periods, potentially effecting N supply to the cultivation system by supplying nutrients from the fish cages or from the nutrient enriched Alexander estuary, is presented in Figure S9.


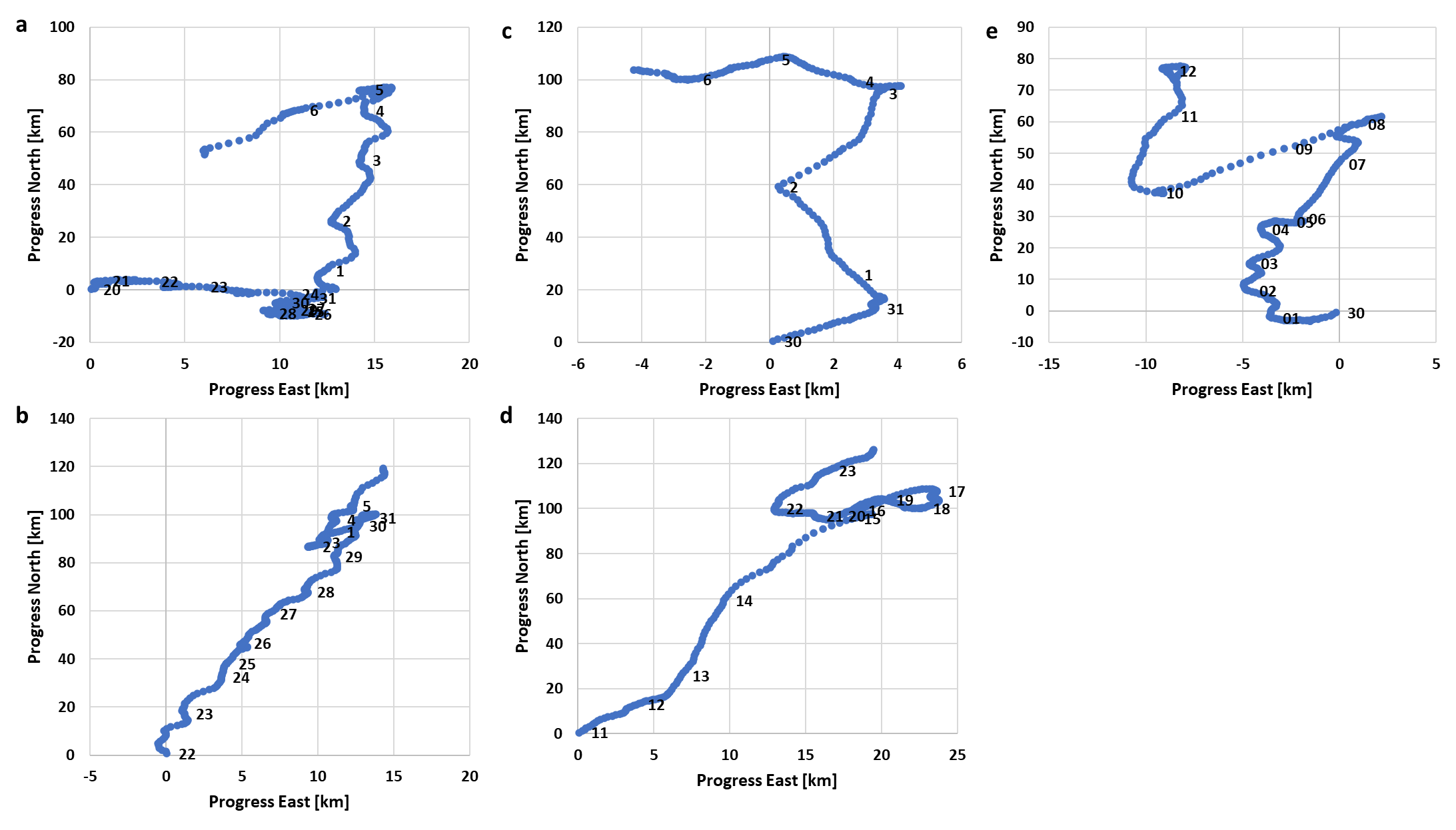


**Figure S9.** Current regime analysis of the preliminary experiment **(a)** and experiments #1 **(b)**, #2 **(c)**, #3 **(d)** and #4 **(e)** of the offshore Ulva sp. cultivation experiment, as measured in the Hadera GLOSS #80 station. Numbers on the plots represent the day of the month. Yellow stars in the preliminary experiment and in experiment #1 represent the harvesting day of the first cultivation period, after continuous fertilizing. The red star in experiment #3 represents the harvesting day of the first half of the cages in experiment #4.
